# Case report: Butylphthalide-associated severe thrombocytopenia, a rare adverse drug reaction

**DOI:** 10.3389/fphar.2026.1903450

**Published:** 2026-09-29

**Authors:** Chenyi Gong, Xiaoling Feng, Yeqing Hu, Jian Wang, Yanan Zhang, Xiaoyan Sun, Bing Zhang, Ying Liu, Hengya Zhu

**Affiliations:** 1 Huzhou Central Hospital, The Affiliated Central Hospital of Huzhou Normal University, Huzhou, Zhejiang, China; 2 The Fifth School of Clinical Medicine, Zhejiang Chinese Medical University, Hangzhou, Zhejiang, China; 3 Huzhou Normal University, Huzhou, China

**Keywords:** butylphthalide, case report, cerebral infarction, drug side effects, thrombocytopenia

## Abstract

**Introduction:**

Butylphthalide (DL-NBP) is a widely used neuroprotective agent for acute ischemic stroke. Although generally well-tolerated, severe thrombocytopenia as a rare adverse reaction has not been fully recognized. Herein, we report a severe case of probable DL-NBP-associated thrombocytopenia to increase clinical awareness and inform clinical management.

**Case Presentation:**

A 69-year-old male with hypertension was admitted for acute brainstem infarction. His hospitalization was complicated by catastrophic thrombocytopenia (min 1.0 × 10^9^/L), suggestive of a possible immune-mediated thrombocytopenia. Subsequent complications included hypoxemia, pneumonia, occult gastrointestinal blood loss, and muscular venous thrombosis of the right calf, creating complex management challenges. Through multidisciplinary collaboration, multimodal tailored treatment was delivered to balance the risks of stroke recurrence and bleeding: corticosteroids, platelet transfusion, recombinant human thrombopoietin and recombinant human interleukin-11 were administered for presumed immune-mediated thrombocytopenia, and antiplatelet therapy was sequentially modified. The patient’s condition stabilized with normalized platelet counts, and he was transferred for rehabilitation.

**Conclusion:**

Severe thrombocytopenia showed a temporal association with DL-NBP treatment. After excluding alternative etiologies, probable DL-NBP-associated thrombocytopenia was considered in this case.

## 1 Introduction

Acute ischemic stroke (AIS) refers to the sudden disruption of cerebral blood perfusion in a localized brain region with various causes. AIS is a major cause of mortality and permanent disability globally. As a catastrophic cerebrovascular disease, AIS leads to over 3.5 million global deaths annually and leaves tens of millions of patients with lifelong functional disability (Feigin et al., 2024). Cerebral reperfusion has been recommended as a standard treatment strategy for AIS (Sharma and Lee, 2025; Ho and Powers, 2025), whereas, approximately half of patients failed to benefit from timely initiation of acute reperfusion therapy (Wang et al., 2023). Prolonged cerebral hypoperfusion inevitably leads to irreversible neuronal cell death. Therefore, effective neuroprotective agents and rehabilitative strategies are urgently needed to rescue damaged neurons and preserve neurological function in clinical practice.

Butylphthalide (DL-NBP) is a common neuroprotective drug for AIS (Xu et al., 2019). DL-NBP acts on multiple links of cerebral ischemia pathology and plays a protective role on cerebral infarction through anti-inflammation, antioxidation, anti-apoptosis, and microcirculation protection (Chang and Wang, 2003; Zhang et al., 2006; Bi et al., 2016; Zhang et al., 2021a; Liu et al., 2021; Xue et al., 2016). Despite its widespread clinical application, the adverse reaction profile of DL-NBP remains incompletely clarified. It has been previously reported to induce elevated liver enzymes and allergic responses (Wang et al., 2022; Wang et al., 2023), while thrombocytopenia as a side effect has not been distinctly confirmed. This article further explores the safety of clinical application of DL-NBP through the diagnosis and treatment process of one patient with acute cerebral infarction who received DL-NBP.

## 2 Case description

A 69-year-old male developed numbness and weakness in the right limb without obvious precipitating factors. After the initial symptoms, the numbness and weakness in the right limb suddenly worsened—he had difficulty lifting the right upper limb and walking with the right lower limb. He was admitted to the emergency department of our hospital 8 h after the onset of the above symptoms. According to his medical records, he had a 7-year history of hypertension and was currently taking felodipine to control blood pressure. Emergency head computed tomography examination showed “possible brainstem infarction”, and he was diagnosed with acute cerebral infarction. Based on vascular imaging findings, the stroke mechanism was classified as large-artery atherosclerosis (Figure 2).

On admission, his vital signs were as follows: body temperature 37.2 °C, blood pressure 191/90 mmHg, pulse rate 110 beats per minute, and respiratory rate 18 breaths per minute. Physical examination revealed right-sided central facial-tongue palsy, right limb hemiplegia, hypoalgesia on the right side of the body, and positive Babinski sign on the right side. His National Institutes of Health Stroke Scale (NIHSS) score was 4 (slight right-sided central facial weakness, grade 4 muscle strength of the right limbs, intact speech and swallowing function), and the Kubota Drinking Test score was Grade 1.

After admission, due to presentation beyond the therapeutic time window, the patient was ineligible for intravenous thrombolysis. Therefore, he received treatment including aspirin enteric-coated tablets for antiplatelet therapy, DL-NBP for neuroprotective therapy, and statins for anti-atherosclerosis. Specifically, after receiving DL-NBP injection on May 12, his platelet count decreased significantly to 10 × 10^9^/L on May 14. DL-NBP was immediately discontinued, and treatment with methylprednisolone and platelet transfusion was initiated. However, the platelet count continued to decrease, dropping to 3 × 10^9^/L in the re-examination on the same day. Although 10 units of platelet concentrates were transfused on May 15 and yielded a transient rise in platelet count, a secondary sharp decline ensued, reaching the nadir of 1 × 10^9^/L on May 20 together with occult gastrointestinal blood loss (fecal occult blood 1+). It should be noted that the subsequent references to occult gastrointestinal blood loss throughout the manuscript are based solely on repeated positive fecal occult-blood tests. No melena, hematochezia, hematemesis, or progressive hemoglobin reduction was observed, and gastrointestinal endoscopy was not performed to confirm a bleeding source. The biphasic fluctuation after transfusion cannot be attributed to a single cause. We hypothesized that this biphasic platelet fluctuation might result from a combination of occult gastrointestinal blood loss and presumed immune-mediated platelet clearance potentially involving drug-dependent platelet-reactive antibodies; however, laboratory assays for such antibodies were not performed in this patient. Moreover, the patient presented with multiple concurrent complications, including hypoxemia, pneumonia, occult gastrointestinal blood loss, and muscular venous thrombosis of the right calf. Thus, emergency endotracheal-intubation assessment and multidisciplinary rescue measures were required.

## 3 Diagnostic assessment

### 3.1 Investigations

After the patient developed a sharp decrease in platelet count and multiple-system dysfunction, the medical team performed a systematic etiological work-up. Blood tests showed characteristic dynamic changes in platelet count: within 48 h after intravenous infusion of DL-NBP, the platelet count dropped sharply to 10 × 10^9^ L on May 14, which might suggest a clinical course of drug-associated immune thrombocytopenia (Vayne et al., 2020); subsequent re-testing on the same day showed a further drop to 3 × 10^9^/L. DL-NBP and aspirin were immediately discontinued and platelet transfusion was initiated on May 14. Even with these interventions, the platelet count dropped to a trough of 1 × 10^9^/L on May 20 (the sixth day after DL-NBP withdrawal), and platelet counts did not recover to 170 × 10^9/^L until June 12. This biphasic decline pattern shares some features reported in cases of drug-associated immune thrombocytopenia, although this finding alone is not pathognomonic for this condition (Bakchoul and Marini, 2018; Arnold et al., 2013).

Bone-marrow examination showed an increased number of megakaryocytes with maturation disorder. While this morphological pattern might be compatible with peripheral platelet-destructive thrombocytopenia, it constituted neither direct evidence of immune-mediated platelet clearance nor specific proof implicating DL-NBP as the causative agent. These bone-marrow features only offered morphological clues indicating that thrombocytopenia was more likely driven by predominant peripheral platelet consumption rather than defective bone-marrow platelet production. This interpretation aligns with the diagnostic work-up framework laid out in the International Consensus Report (Provan et al., 2019).

The treatment response provided further supportive clinical clues for probable DL-NBP-associated thrombocytopenia, rather than definitive proof of causation. After discontinuing DL-NBP, the patient experienced a second platelet collapse on May 20; nevertheless, the platelet counts eventually demonstrated a steady upward trend following intravenous treatment with methylprednisolone and supportive platelet transfusion. Meanwhile, the temporal correlation between the adjustment of antiplatelet drugs (aspirin discontinued on May 14, clopidogrel initiated on May 30, switched to cilostazol on June 3) and platelet fluctuations was poor, and no recurrent thrombocytopenia occurred after cilostazol initiation. These observations further rendered a major contributory effect for antiplatelet agents unlikely. Collectively, the case was clinically suspected to represent probable DL-NBP-associated thrombocytopenia.

Diagnoses of hypoxemia, pneumonia, occult gastrointestinal blood loss and right-calf muscular venous thrombosis were primarily confirmed by laboratory tests and imaging examinations. An arterial partial pressure of oxygen<60 mmHg (PaO_2_ = 52.8 mmHg) on arterial blood gas analysis indicated hypoxemia. The diagnosis of pneumonia was established based on comprehensive multi-faceted assessments. Chest computed tomography revealed patchy pulmonary infiltrates (Figure 3); laboratory tests demonstrated elevated white blood cell count (13.9 × 10^9^/L) and neutrophil count on routine blood examination (11.6 × 10^9^/L), accompanied by increased C-reactive protein (41.2 mg/L). Collectively, multiple laboratory and imaging findings supported the complex clinical manifestations in this patient. Table 1 summarizes the complete chronological course of platelet count fluctuations, pharmacotherapeutic interventions, and key clinical events throughout the hospital stay, which provides an overview of the patient’s disease trajectory before detailed treatment description in the following section.

**TABLE 1:** Timeline of platelet counts, key therapeutic interventions, and clinical events. This table chronologically documents the patient’s platelet counts, corresponding major treatments and occult gastrointestinal blood loss status from May 12 to 16 June 2019. It highlights the catastrophic drop in platelet levels following the onset of symptoms and the subsequent multidisciplinary management, including the critical decision to discontinue butylphthalide, platelet transfusions, initiation of high-dose corticosteroids (methylprednisolone), and the stepwise adjustment of antiplatelet therapy in response to the patient’s evolving clinical status.

Date	Platelet count (PLT, ×10^9^/L)	Medication and key events
2019–05-12	225.0	Aspirin enteric-coated tablets, butylphthalide, statin tablets, citicoline injection, xueshuantong
2019–05-14	10.0→3.0	Butylphthalide discontinued, platelet transfusion (10 units), Interleukin-11 injection, methylprednisolone 80 mg IV
2019–05-15	5.0	Continued platelet transfusion (10 units), Interleukin-11 injection, moxifloxacin (anti-infection), methylprednisolone 80 mg IV
2019–05-16	9.0	Interleukin-11 injection, methylprednisolone 40 mg IV
2019–05-17	4.0	Interleukin-11 injection, methylprednisolone 40 mg IV
2019–05-18	Unchecked	Interleukin-11 injection, methylprednisolone 40 mg IV
2019–05-19	32.0	Interleukin-11 injection, methylprednisolone 40 mg IV
2019–05-20	1.0	Emergency crossmatch for platelets (10 units), recombinant human thrombopoietin, Interleukin-11 injection, methylprednisolone 120 mg IV, fecal occult blood 1+
2019–05-21	52.0	Methylprednisolone 80 mg IV
2019–05-22	22.0	Platelet transfusion (10 units), meropenem (for anti-infection), methylprednisolone (60 mg IV), fecal occult blood negative
2019–05-23	45.0	Methylprednisolone 40 mg IV
2019–05-24	131.0	Methylprednisolone 40 mg IV
2019–05-25	176.0	Methylprednisolone 40 mg IV
2019–05-26	271.0	Methylprednisolone 40 mg IV
2019–05-27	271.0	Methylprednisolone tapered to 30 mg IV
2019–05-28	298.0	Methylprednisolone 30 mg IV
2019–05-30	321.0	Clopidogrel (antiplatelet), rabeprazole (acid suppression and gastric protection), other treatments continued
2019–06-01	325.0	Medrol 5 tablets/day (to elevate platelets), other treatments continued
2019–06-03	273.0	Clopidogrel discontinued, calamine lotion, pevisone cream, mizolastine tablets added, cilostazol (antiplatelet) added, medrol 5 tablets/day
2019–06-04	Unchecked	Sodium heparin 4000 IU (0.4 mL), other treatments continued
2019–06-05	219.0	Medrol tapered to 4 tablets/day, sodium heparin 4000 IU (0.4 mL), other treatments continued, fecal occult blood negative
2019–06-06	Unchecked	Sodium heparin 4000 IU (0.4 mL) (discontinued), fecal occult blood 2+, other treatments continued
2019–06-07	206.0	Fecal occult blood 2+, other treatments continued
2019–06-08	200.0	Fecal occult blood 3+, other treatments continued
2019–06-09	Unchecked	Fecal occult blood 2+, other treatments continued
2019–06-10	Unchecked	Fecal occult blood 1+, other treatments continued
2019–06-11	Unchecked	Fecal occult blood weakly positive, other treatments continued
2019–06-12	170.0	Human albumin injection 50 mL IV, other treatments continued
2019–06-16	219.0	Transferred to rehabilitation with ongoing cilostazol antiplatelet therapy

Fecal occult-blood testing was positive on May 20. No stool occult-blood laboratory assessments were performed between May 21 and June 5. Testing was repeated on June 6, when a positive fecal occult-blood test recurred, without progressive hemoglobin reduction.

### 3.2 Treatment

On May 12, the patient was admitted for AIS and received routine therapy with aspirin enteric-coated tablets, DL-NBP, statin tablets, and citicoline injection. On May 13, the patient’s right limb weakness worsened and dysphagia occurred. A nasogastric tube was inserted for feeding, and the treatment plan remained unchanged. On May 14, the patient developed chest tightness and decreased blood oxygen saturation, and pulmonary infection was considered. Ceftriaxone sodium was added for anti-infection treatment, and measures such as oxygen inhalation, sputum suction, and nebulization were given. Methylprednisolone 80 mg intravenously was added during the emergency management. After the platelet count was first reported as a critical value of 10 × 10^9^/L, intervention was immediately initiated: permanent discontinuation of DL-NBP; due to the progressive decrease in platelet count, aspirin was discontinued simultaneously. In addition, platelet transfusion and recombinant human interleukin-11 injection were administered for platelet elevation.

On May 15, platelet transfusion and recombinant human interleukin-11 injection were continued, methylprednisolone was maintained at 80 mg intravenously, and moxifloxacin was initiated to strengthen anti-infection treatment. On May 16, the dosage of methylprednisolone was adjusted to 40 mg intravenously, and recombinant human interleukin-11 was given to elevate platelets. This thrombopoietic agent was administered from May 14 to May 19. On May 20, the patient developed hiccups and occult gastrointestinal blood loss; acid-suppressive treatment was given, platelet transfusion was performed again, and bone marrow aspiration was conducted. A single dose of recombinant human thrombopoietin (rhTPO) was administered on May 20. Meanwhile, intravenous methylprednisolone was escalated to 120 mg as the platelet count dropped to a nadir of 1 × 10^9^/L. On May 21, methylprednisolone was adjusted to 80 mg intravenously given the gradual recovery of platelet count. On May 22, several antibiotics were discontinued, meropenem was initiated instead, and the dosage of methylprednisolone was adjusted to 60 mg intravenously. Subsequently, the dosage of methylprednisolone was titrated according to the patient’s condition (40 mg intravenously on May 23, 30 mg intravenously on May 27, oral methylprednisolone on June 1), and related complications were managed. On May 30, no recent positive fecal occult-blood test results were available, and bleeding risk was judged to be low on clinical grounds; clopidogrel was initiated after platelet count normalization. On June 3, owing to rash developing during clopidogrel administration, clopidogrel was discontinued and replaced with cilostazol. Meanwhile, given persistent weakness of the patient’s right lower limb and increased D-dimer, venous color-Doppler ultrasonography was performed for thrombotic screening, which revealed muscular venous thrombosis localized to the right calf. Despite the complex clinical background including AIS, severe thrombocytopenia, ongoing occult gastrointestinal blood loss, prior antiplatelet exposure, and administration of thrombopoietic agents, therapeutic heparin anticoagulation was initiated after careful risk-benefit evaluation of bleeding *versus* thrombotic hazards. The anticoagulation regimen consisted of subcutaneous sodium heparin injection at a dose of 4,000 IU (0.4 mL), administered once daily, from June 4 to June 6, initiated when the platelet count was 273 × 10^9^/L on June 3. It was discontinued on June 6 because of concerns regarding persistent occult gastrointestinal blood loss and declining D-dimer levels. On June 16, the patient was scheduled for transfer to another hospital for hyperbaric oxygen therapy and rehabilitation exercises.

### 3.3 Outcome and follow-up

After comprehensive multidisciplinary treatment, the patient’s vital signs remained stable. Although stroke progression occurred in the early inpatient period and neurological deficits peaked at an NIHSS score of 9, subsequent intervention halted further deterioration of neurological function: the patient’s limb weakness no longer worsened, with right upper limb muscle strength stabilized at Grade 2+ and right lower limb strength at Grade 3. Persistent manifestations included right-sided pinprick hypoesthesia and a positive Babinski sign on the right.

Remarkable improvements were achieved for the acute life-threatening complications: pulmonary infection was fully controlled with sustained normal blood-oxygen saturation; platelet count returned to the normal range (Figure 1), (Figure 2; Figure 3). cutaneous hemorrhagic petechiae completely resolved, and occult gastrointestinal blood loss was brought under control. Nevertheless, residual dysarthria persisted.

**FIGURE 1:**
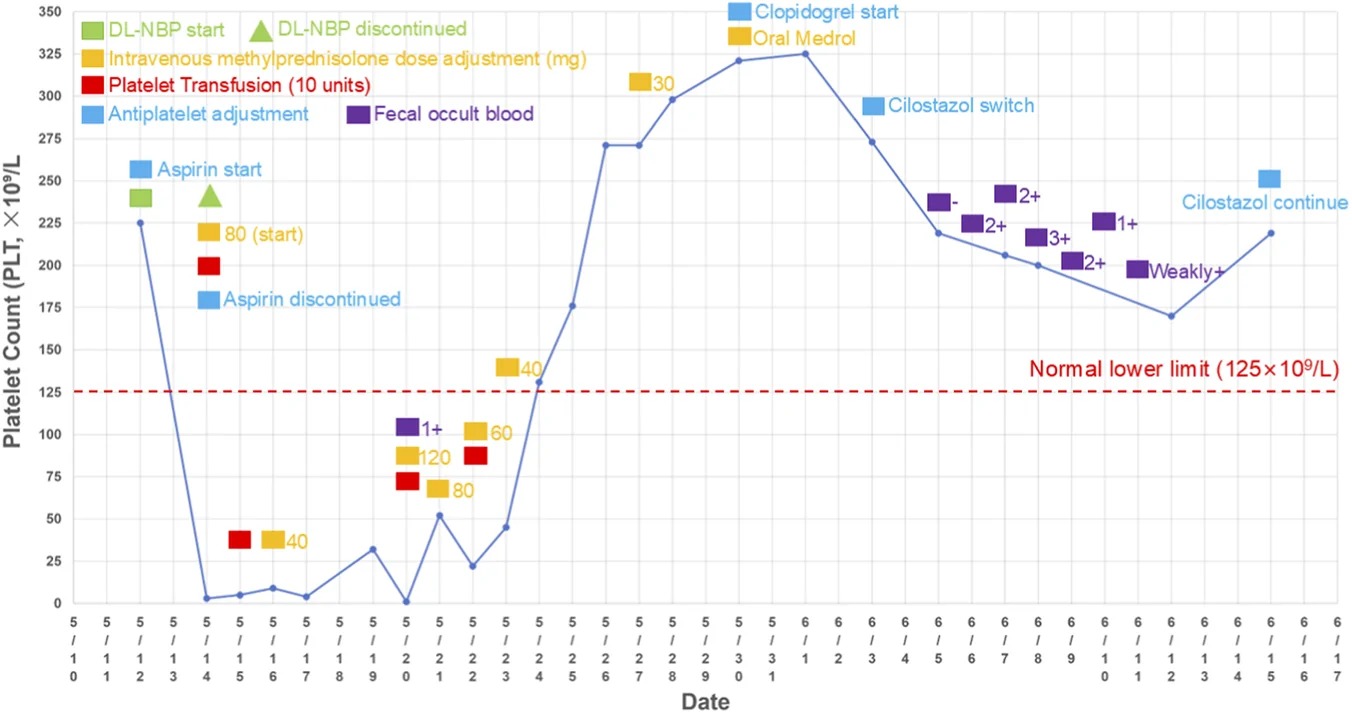
Serial dynamic changes in platelet count and key clinical events throughout hospitalization. The solid blue line represents daily platelet count. The red dashed horizontal line marks the lower normal threshold for platelet count (125 ×10^9^/L). Green square: initiation of DL-NBP; green triangle: discontinuation of DL-NBP; yellow square: intravenous methylprednisolone dose adjustment; red square: platelet transfusion; blue square: antiplatelet agent initiation, discontinuation or switching; purple square: results of fecal occult blood test. After DL-NBP administration, the platelet count dropped sharply, followed by a biphasic fluctuation. Gradual platelet recovery was observed after discontinuation of DL-NBP, glucocorticoid therapy and platelet transfusion. Recurrent positive fecal occult blood indicated persistent gastrointestinal bleeding during the later hospitalization period. DL-NBP: Butylphthalide.

**FIGURE 2:**
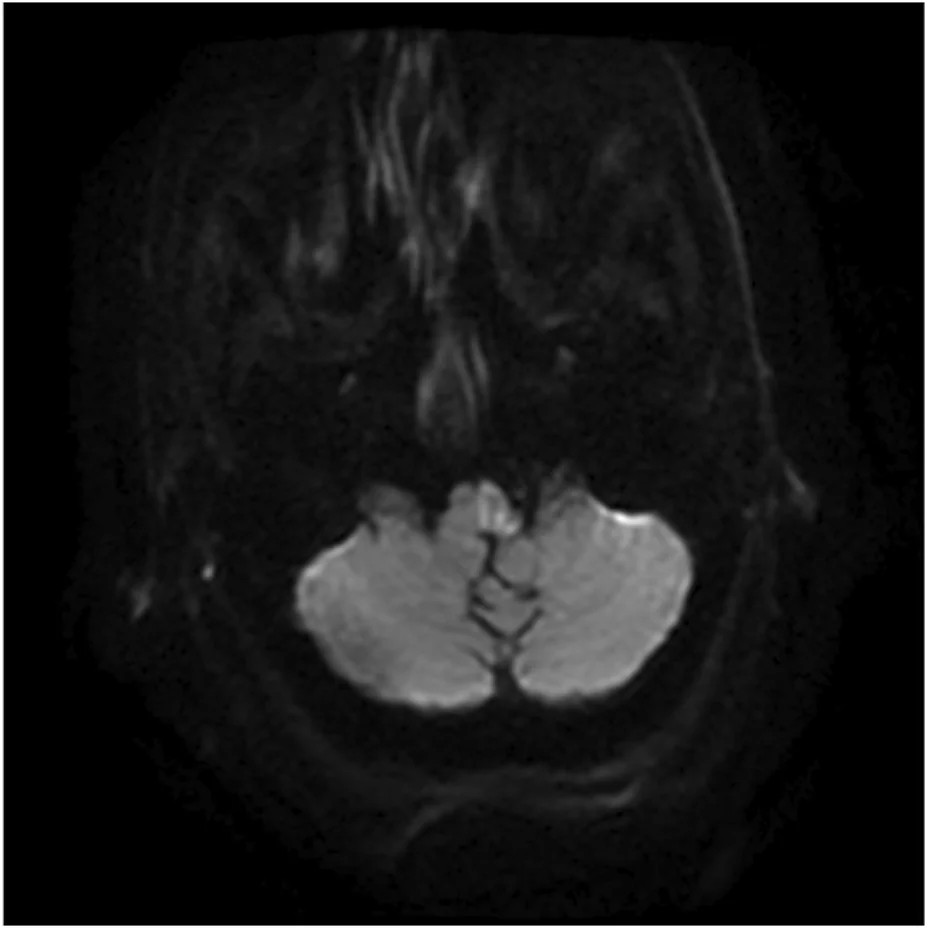
Representative brain magnetic resonance imaging images revealing acute brainstem infarction.

**FIGURE 3:**
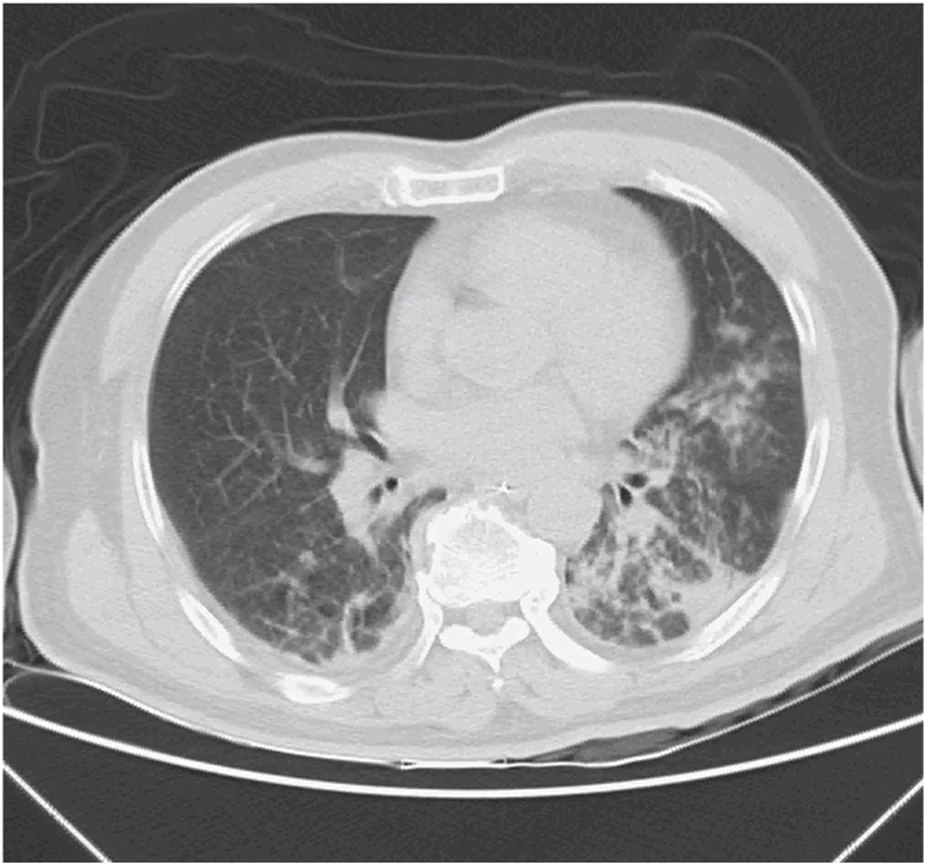
Chest computed tomography scans showing signs of pulmonary infection.

After discharge, the patient required continuation of baseline medications, regular monitoring of complete blood count, liver and kidney function, and other laboratory parameters, intensive limb rehabilitation training, aspiration precautions, and scheduled outpatient follow-up.

## 4 Discussion

This case presented severe thrombocytopenia occurring in a 69-year-old male patient with AIS who received DL-NBP therapy. As a commonly used drug for AIS, the safety profile of DL-NBP has been well-documented (Wang et al., 2023; Zhang et al., 2021b; Fan et al., 2021). Previous large-scale clinical studies have demonstrated that its adverse reactions predominantly involve the digestive system (such as nausea and vomiting, with an incidence of 5%–15%) and liver function abnormalities (elevated liver enzymes, with an incidence of 10%–20%); the incidence of neurological symptoms such as dizziness and headache (Wang et al., 2022) is about 3%–8%. However, to our knowledge, severe thrombocytopenia associated with DL-NBP has been rarely reported. Observations from our case provide clinical clues for probable DL-NBP-associated severe thrombocytopenia and further complement the safety data for the clinical application of this agent. The onset, clinical course, and management of this adverse event may offer preliminary references for the safe clinical use of DL-NBP.

From the perspective of temporal correlation analysis, the patient’s thrombocytopenia showed features suggestive of drug-associated thrombocytopenia. Within 48 h after intravenous infusion of DL-NBP, the platelet count dropped sharply to 10 × 10^9^/L. Although methylprednisolone 80 mg intravenously was initiated, the patient’s platelet count continued to decline. Despite the temporary increase in the platelet count after platelet transfusion, it still deteriorated to a nadir of 1 × 10^9^/L on May 20. This biphasic decline pattern shared some clinical features of drug-associated immune thrombocytopenia, rather than direct bone marrow toxicity. Direct bone marrow toxicity usually leads to gradual thrombocytopenia and steady recovery after drug cessation, without sustained peripheral platelet destruction or a biphasic post-transfusion course. By contrast, this biphasic change was more suggestive of drug-associated thrombocytopenia (Bakchoul and Marini, 2018). One possible hypothetical underlying mechanism involves circulating drug-dependent antibodies capable of clearing both autologous and transfused platelets, though no such testing was performed to verify this hypothesis in the present case. For drug-associated immune thrombocytopenia, pathogenesis is predominantly explained by the hapten hypothesis: small-molecule drugs such as DL-NBP act as haptens, binding covalently to platelet-membrane proteins to form novel immunogenic complexes, which trigger the production of drug-dependent anti-platelet antibodies (George and Aster, 2009).

This kinetic profile stemmed from multiple interacting factors: one hypothetical contributing factor was a putative mechanism involving circulating drug-dependent anti-platelet antibodies that might clear both autologous and transfused platelets, and another factor was occult gastrointestinal blood loss which may have caused extra platelet consumption and blunted the post-transfusion platelet increment. The platelet count reached its trough 6 days after DL-NBP withdrawal and then recovered gradually under comprehensive treatment. This overall dynamic change provided clinical clues for a probable immune-mediated pathogenesis for DL-NBP-associated thrombocytopenia and added to clinical awareness of its rare hematologic adverse reactions.

The gradual rebound of platelets stemmed from several combined interventions: DL-NBP withdrawal, immunosuppression by methylprednisolone, and platelet production promotion by interleukin-11 and rhTPO. Interleukin-11 binds to interleukin-11 receptor-α on megakaryocyte progenitors, activates the downstream gp130/STAT3 signaling cascade, and directly drives megakaryocyte proliferation and terminal maturation; rhTPO activates mitogen-activated protein kinase pathways to regulate the process of megakaryocytopoiesis (Rojnuckarin et al., 1999). Both interleukin-11 and rhTPO stimulate megakaryocyte proliferation and elevate peripheral platelet counts, while their thrombopoietic activity carries inherent prothrombotic risk. Therefore, in this patient complicated with muscular venous thrombosis of the right calf, we performed rigorous risk-benefit stratification balancing recurrent stroke, bleeding and thrombotic events before initiating heparin anticoagulation. This case underscores the necessity of individualized management to balance bleeding and thrombotic risks during thrombopoietic therapy.

In terms of diagnosis, the strength of this case lies in the application of a multi-dimensional evaluation. The bone-marrow examination showed an increased number of megakaryocytes with maturation disorder, which yielded morphological clues that might point toward peripheral-driven platelet destruction, without proving immune-mediated mechanisms. By dynamically monitoring the treatment response and conducting treatment in accordance with the American Society of Hematology 2019 Guidelines for Immune Thrombocytopenia (Neunert et al., 2019), we observed clinical improvement after combined therapy with glucocorticoids, platelet transfusion, interleukin-11, and rhTPO. This comprehensive clinical evaluation strategy, which integrates multiple lines of evidence rather than relying solely on therapeutic response, is not only applicable to the assessment of DL-NBP-associated thrombocytopenia but also offers a referential clinical strategy for identifying adverse reactions associated with other novel agents.

Regarding the differential diagnosis, probable DL-NBP-associated thrombocytopenia should be distinguished from pseudothrombocytopenia, sepsis/infection-associated thrombocytopenia, disseminated intravascular coagulation (DIC), thrombotic thrombocytopenic purpura/thrombotic microangiopathy (TTP/TMA), heparin-induced thrombocytopenia (HIT), viral infection, autoimmune disease, liver disease or splenomegaly.

Pseudothrombocytopenia describes a falsely low platelet count resulting from *in vitro* platelet aggregation (Cattaneo, 2025). No platelet clumping was flagged on complete blood count analysis, and the mean platelet volume was largely within the reference range (9.1 fL). Notably, a manual peripheral-blood smear for evaluation of platelet clumping and schistocytes, as well as confirmatory testing using citrate-anticoagulated blood tubes were not performed in this patient. In addition, the patient presented with diffuse cutaneous petechiae, consistent with true severe thrombocytopenia. Taken together, pseudothrombocytopenia was considered unlikely. Sepsis/infection-associated thrombocytopenia was considered. However, there was no evidence of systemic inflammatory decompensation to support sepsis, making infection-driven thrombocytopenia unlikely. DIC is characterized by consumptive coagulopathy, typically manifesting as prolonged prothrombin time and activated partial thromboplastin time, alongside elevated D-dimer (L et al., 2009). Serial coagulation assays in this patient remained largely normal, arguing against DIC (prothrombin time: 12.0 s, activated partial thromboplastin time: 18.4 s; D-dimer: 0.21 mg/L). Isolated thrombocytopenia was the only hematological abnormality observed in this case. Laboratory findings showed no evidence of microangiopathic hemolysis, with hemoglobin of 145 g/L and lactate dehydrogenase of 201.6 U/L. Nevertheless, in the absence of a manual peripheral-blood smear, TTP/TMA can only be deemed improbable and cannot be definitively ruled out (George and Nester, 2014; Chiasakul and Cuker, 2018). The patient had no heparin exposure during the period of platelet-count decline, rendering a diagnosis of HIT unsupported. For viral infection, the virological screening of this patient revealed no evidence of active infection, making virally mediated thrombocytopenia less probable. All autoantibody assays returned negative, rendering thrombocytopenia secondary to autoimmune disorders unlikely. Furthermore, liver function tests and hepatic-splenic imaging demonstrated no abnormalities, rendering thrombocytopenia attributable to liver disease or splenomegaly improbable. Representative laboratory parameters supporting these differential-diagnosis assessments are summarized in Supplementary Table S1.

After excluding the above alternative etiologies, we further applied the Naranjo Adverse Drug Reaction Probability Scale to assess the likelihood of thrombocytopenia being an adverse reaction to each administered drug (Table 1), (Table 2) (Naranjo et al., 1981). Causality grading for each drug was assigned according to the total score based on the original Naranjo criteria. DL-NBP achieved a total score of 6, corresponding to a Probable causality grade. Aspirin was scored two points (Possible causality), whereas statin tablets, citicoline injection and Xueshuantong were scored three points each (Possible causality). Antibiotics, gastric-protective agents, clopidogrel and cilostazol were initiated after thrombocytopenia onset, yielding total scores of −1 and classified as Doubtful causality. Notably, platelet recovery occurred under multiple concurrent therapeutic interventions rather than DL-NBP withdrawal alone. Although Naranjo scale scoring identified DL-NBP as the most plausible suspect agent associated with this patient’s thrombocytopenia, this assessment only supports a probable association and cannot constitute definitive proof of causality.

**TABLE 2:** Scores of the Naranjo Adverse Drug Reaction Probability Scale for all administered medications.

Medications	Butyphthalide	Aspirin	Statin therapy	Citicoline	Xueshuantong	Antibiotics	Gastric-protective treatment	Clopidogrel	Cilostazol
1. Are there previous conclusive reports on this reaction?	0	+1	+1	+1	+1	+1	+1	+1	+1
2. Did the adverse event appear after the suspected drug was administered?	+2	+2	+2	+2	+2	−1	−1	−1	−1
3. Did the adverse reaction improve when the drug was discontinued or a specific antagonist was administered?	+1	0	0	0	0	0	0	0	0
4. Did the adverse reaction reappear when the drug was readministered?	0	−1	0	0	0	0	0	0	0
5. Are there alternative causes (other than the drug) that could on their own have caused the reaction?	+2	−1	−1	−1	−1	−1	−1	−1	−1
6. Did the reaction reappear when a placebo was given?	0	0	0	0	0	0	0	0	0
7. Was the drug detected in the blood (or other fluids) in concentrations known to be toxic?	0	0	0	0	0	0	0	0	0
8. Was the reaction more severe when the dose was increased, or less severe when the dose was decreased?	0	0	0	0	0	0	0	0	0
9. Did the patient have a similar reaction to the same or similar drugs in any previous exposure?	0	0	0	0	0	0	0	0	0
10. Was the adverse event confirmed by any objective evidence?	+1	+1	+1	+1	+1	0	0	0	0
Total score	6	2	3	3	3	−1	−1	−1	−1
Causality grade	Probable	Possible	Possible	Possible	Possible	Doubtful	Doubtful	Doubtful	Doubtful

Causality grades were classified according to the original Naranjo criteria: total score ≥9: Definite; five to 8: Probable; one to 4: Possible; total score ≤0: Doubtful. for aspirin, rechallenge occurred during post-discharge follow-up; aspirin was re-administered, and thrombocytopenia did not recur; hence question four was scored −1.

It is worth noting that although management measures generally considered for probable drug-associated thrombocytopenia (including discontinuation of the suspected offending agent, glucocorticoid administration, among others) were implemented in this case, this patient with acute cerebral infarction encountered distinctive clinical challenges: balancing suppression of the presumed immune-mediated platelet clearance against the requirement for adequate antiplatelet prophylaxis to avert stroke progression. These competing clinical priorities prompted the adoption of a refined treatment strategy tailored to this individual patient and different from the conventional one.

The optimization of the treatment strategy is an important insight from this case. The first priority is to immediately discontinue the suspected drug, which was done in this case as soon as thrombocytopenia was detected. In terms of drug selection, intravenous methylprednisolone was given with the aim of suppressing the putative immune-mediated pathological process. Recombinant human interleukin-11, which is known to promote megakaryocyte maturation and platelet production, was administered early in the clinical course; and platelet transfusion provided protection against the risk of severe bleeding. Moreover, the adjustment of antiplatelet drugs took into account both the necessity of thrombosis prevention and the potential impact of drugs on platelets, demonstrating the refinement of clinical decision-making. Compared with conventional thrombocytopenia treatment, these differentiated management methods ensured the antithrombotic effect while minimizing the bleeding risk as much as possible.

The particularity of this case lies in the fact that, as a stroke patient, the management of thrombocytopenia involved more complex clinical trade-offs, and clinical decisions were made in accordance with the Guidelines for the Early Management of Patients With Acute Ischemic Stroke (Powers et al., 2019). On the one hand, it was necessary to maintain antiplatelet therapy to prevent stroke progression; on the other hand, it was necessary to address the bleeding risk caused by thrombocytopenia. Such therapeutic dilemma is particularly prominent in patients with probable drug-associated thrombocytopenia complicated by acute ischemic stroke. The clinical course observed in this case may offer preliminary clinical insights for similar complex clinical scenarios.

Compared with previous reports, this case report describes a full-process management framework covering “diagnosis-treatment-prognosis” for the clinical challenge of cerebral infarction complicated with thrombocytopenia: drug-adverse reaction association was evaluated via temporal-sequence analysis; a stepwise antiplatelet regimen was applied to balance thrombotic and bleeding risks; and neurological function assessment was integrated into treatment-response monitoring. The observations from this case may provide practical references for managing similar clinical scenarios. From a drug-safety perspective, although adverse reactions listed in the DL-NBP package insert are predominantly liver-enzyme elevation and allergic manifestations, the present case suggests that DL-NBP may be associated with severe thrombocytopenia. Clinicians should closely monitor platelet counts during administration of this agent, particularly in the early treatment phase. Increased vigilance is needed for patients with prior drug allergy or autoimmune disease, and preventive measures should follow the recommendations in the 2021 Guideline for the Prevention of Stroke in Patients With Stroke and Transient Ischemic Attack (Kleindorfer et al., 2021).

This case has several limitations. First, drug-dependent platelet antibody assays were not performed; thus, direct laboratory evidence for DL-NBP-associated anti-platelet antibodies is lacking. Of note, this assay has relatively low analytical sensitivity, and discontinuation of the suspected offending agent represents standard clinical management (Bakchoul and Marini, 2018). A negative antibody result cannot fully rule out probable drug-associated thrombocytopenia, whereas a positive DL-NBP-dependent platelet antibody finding would substantially strengthen causal inference. Second, the patient had no documented prior exposure to DL-NBP. The rapid platelet decline occurring within approximately 48 h is inconsistent with the classic time-course pattern of drug-associated immune thrombocytopenia following first-time drug exposure. Nevertheless, atypical temporal presentations have been occasionally reported in the literature (Bhaskarla et al., 2022). This temporal feature alone cannot either confirm or completely rule out a probable association with DL-NBP. Therefore, causality cannot be judged based on the disease course in isolation, and comprehensive multi-faceted assessment remains essential for causality evaluation. Importantly, complete retrospective reconstruction of every prior medication is not always feasible, so unrecorded prior-drug exposure cannot be definitively excluded. Third, manual peripheral-blood smear examination was not performed. Although the complete blood count analyzer did not flag obvious platelet clumping, automated hematology analyzer alerts cannot substitute manual microscopic review. The absence of this test precluded direct visual assessment for platelet clumping (to rule out pseudothrombocytopenia) and schistocytes (features suggestive of TMA-related hemolysis), leaving these differential diagnoses not fully excluded. Fourth, the Naranjo scale is a clinical probability-assessment tool rather than a confirmatory laboratory test. Fifth, gastrointestinal endoscopy was not undertaken to identify potential bleeding sources, due to the patient’s critical clinical condition during hospitalization. Therefore, we could not definitively verify whether true gastrointestinal bleeding existed, and the designation of occult gastrointestinal blood loss relied only on repeatedly positive fecal occult-blood test results. Finally, the generalizability of observations derived from a single case report is limited.

## 5 Patient perspective

Now, I have been discharged from the hospital and am continuing ongoing physical therapy and functional training at a rehabilitation facility. Although strength and coordination on my right side have not fully returned, and I still need to persist with daily exercises, I am already able to walk with some support from my family and can eat and carry out some simple daily activities on my own. The bleeding spots on my body have long since faded, and my platelet count has returned to normal-I no longer have to live in constant fear. I know that a full recovery is still a long road ahead, but I am deeply grateful to have come this far. My mindset has gradually calmed; I’ve learned to accept life with this illness and now cherish every moment spent with my family more than ever.

Although I still require rehabilitative training, I am wholeheartedly thankful to the medical team for never giving up on me. With their professional expertise, they found the optimal treatment plan at the brink of life and death, giving me a new lease on life. This experience has made me deeply understand that collaboration and mutual trust between patients and doctors are the key to overcoming disease.

## 6 Conclusion

In conclusion, DL-NBP showed a temporal association with severe thrombocytopenia in this case and should be regarded as a probable suspect agent after excluding alternative etiologies, which underscores the need for close platelet count monitoring during its clinical administration. The diagnosis of drug-related thrombocytopenia relies on comprehensive assessment of temporal relationship, clinical manifestations, laboratory results and treatment response. For stroke patients complicated by thrombocytopenia of suspected drug-related origin, antiplatelet therapy requires individualized adjustment to balance thrombotic and bleeding risks. In this case, platelet recovery occurred following withdrawal of the suspected drug and multimodal treatment.

## Data Availability

The original contributions presented in the study are included in the article/Supplementary Material, further inquiries can be directed to the corresponding authors.
