# Supplementary material for: Case report: Butylphthalide-associated severe thrombocytopenia, a rare adverse drug reaction
Source: Front Pharmacol. 2026 Sep 29;17:1903450. doi: 10.3389/fphar.2026.1903450 (PMC13659440; doi:10.3389/fphar.2026.1903450)
Supplement: Supplementary file 1 [file Table1.docx]

**Supplementary Table 1. Representative laboratory parameters for differential diagnosis of thrombocytopenia**

| Project name | Result | Unit | Reference ranges |
| --- | --- | --- | --- |
| Exclude disseminated intravascular coagulation | | | |
| Activated partial thromboplastin time | 18.4 | s | 22.0-36.0 |
| Prothrombin time | 12.0 | s | 11.0-14.3 |
| D-dimer | 0.21 | mg/L | <0.50 |
| Fibrinogen | 3.50 | g/L | 2.00-4.00 |
| Exclude thrombotic thrombocytopenic purpura/thrombotic microangiopathy | | | |
| Hemoglobin | 145.0 | g/L | 130.0-175.0 |
| Lactic dehydrogenase | 201.6 | U/L | <248 |
| Total bilirubin | 21.7 | μmol/L | 1.7-22.0 |
| Indirect bilirubin | 9.5 | μmol/L | 0.0-17.0 |
| Exclude infection-sepsis-related thrombocytopenia | | | |
| White blood cell count | 13.5×10^9^ | g/L | 3.5-9.5 |
| Neutrophil count | 11.6×10^9^ | g/L | 1.8-6.3 |
| C‑reactive protein | 41.2 | mg/L | 0-10.0 |
| Excluded platelet reduction caused by viruses | | | |
| Hepatitis B surface antigen | 0.00 | IU/mL | 0.00-0.05 |
| Hepatitis C antibody | Negative |  |  |
| Human immunodeficiency virus (1+2) antibody + p24 antigen | Negative |  |  |
| Exclude thrombocytopenia caused by autoimmune diseases | | | |
| Anti-mitochondrial antibody | Negative |  |  |
| Anti-mitochondrial (M2) antibody | Negative |  |  |
| Anti-nuclear antibody | Negative |  |  |
| Anti-centromere antibody | Negative |  |  |
| Anti-ribosomal P protein antibody | Negative |  |  |
| Anti-nucleosome antibody | Negative |  |  |
| Anti-Jo-1 | Negative |  |  |
| Anti-PCNA antibody | Negative |  |  |
| Anti-PM-Scl | Negative |  |  |
| Anti-Ro-52 antibody | Negative |  |  |
| Anti-Scl-70 | Negative |  |  |
| Anti-Sm | Negative |  |  |
| Anti-SSA | Negative |  |  |
| Anti-SSB | Negative |  |  |
| Anti-U1RNP | Negative |  |  |
| Anti-histone antibody | Negative |  |  |
| Anti-double-stranded-DNA | Negative |  |  |
| Exclude thrombocytopenia related to liver disease/hypersplenism | | | |
| Alanine aminotransferase | 29.0 | U/L | 9.0-50.0 |
| Aspartate aminotransferase | 24.0 | U/L | 15.0-40.0 |
| Total bilirubin | 21.7 | μmol/L | 1.7-22.0 |
| Creatinine | 69.6 | μmol/L | 44.0-132.6 |
| Urea nitrogen | 5.49 | μmol/L | 3.20-7.14 |

Representative laboratory values collected during the low‑platelet‑count phase are summarized to evaluate alternative etiologies of thrombocytopenia. Elevated white blood cell count, neutrophil count and C‑reactive protein were consistent with concurrent pulmonary infection, without laboratory evidence of sepsis‑driven consumptive coagulopathy. Manual peripheral‑blood smear and drug‑dependent platelet‑reactive antibodies assays were not conducted in this case.
